# Usutu Virus Sequences in *Culex pipiens* (Diptera: *Culicidae*), Spain

**DOI:** 10.3201/eid1405.071577

**Published:** 2008-05

**Authors:** Núria Busquets, Anna Alba, Alberto Allepuz, Carles Aranda, José Ignacio Nuñez

**Affiliations:** * Centre de Recerca en Sanitat Animal, Barcelona, Spain; † Universitat Autònoma de Barcelona, Barcelona, Spain

**Keywords:** arboviruses infections, flavivirus, Culex, letter

**To the Editor:** *Usutu* *virus *(USUV) is an arbovirus and a member of the mosquito-borne cluster within the *Flavivirus* genus. USUV belongs to the *Japanese encephalitis virus* antigenic group, which is closely related to pathogens such as *West Nile virus* (WNV) (*1*).

USUV has been isolated from a human in the Central African Republic and from several mosquito species from tropical and subtropical Africa (*2*). In late summer 2001, USUV emerged in central Europe and caused deaths in several species of resident birds in Austria (*3*). However, monitoring of USUV in dead birds from 2003 through 2005 showed that the absolute numbers of USUV–associated bird deaths declined, although USUV detection persisted in bird tissues (*4*). This decrease in USUV-associated bird deaths was attributed to herd immunity in the bird population (*5*). In the summer of 2005, USUV was detected in a blackbird in Hungary. The complete genomic sequence of the Hungarian USUV strain shared 99.9% identity with the strain circulating in Austria since 2001 (*6*). On the other hand, neutralizing antibodies against USUV have been detected in sera of resident and migrant birds in the United Kingdom without causing an obvious reduction in the bird population (*7*).

From May through October 2006, monitoring of flaviviruses in mosquitoes was performed in the northeast region of Spain (Catalonia). This monitoring was implemented in the 3 main wetlands of the region: Aigüamolls de la Empordà (Girona Province) near France, where WNV was detected in dead horses in 2000 (*8*); Delta del Llobregat (Barcelona Province); and Delta de l’Ebre (Tarragona Province). Mosquitoes were collected by mosquito control services in these areas. Female mosquitoes were classified and grouped in pools according to date, species, and localization. During this period, 436 pools belonging to 9 mosquito species were collected. The most abundant species was *Culex pipiens* (n = 168).

Viral RNA was recovered from mosquito pools by homogenization and viral RNA extraction with QIAamp Viral RNA Mini Kit (QIAGEN, Valencia, CA, USA), and then generic reverse transcription (RT)-nested PCR was used to identify flaviviruses (*9*). This procedure was used to amplify a specific fragment of the NS5 gene within the flavivirus genome. The 143-bp amplification product was detected by electrophoresis and purified by using QIAquick PCR Purification Kit (QIAGEN). Sequencing reactions were performed with ABI Prism BigDye Terminator Cycle Sequencing v.3.1 Ready Reaction (Applied Biosystems, Foster City, CA, USA), and analyzed by using an ABI PRISM model 3730 automated sequencer (Applied Biosystems). Assembly of the consensus sequences and translation into amino acid sequences was performed with Larsergene DNASTAR group of programs (DNASTAR Inc., Madison, WI, USA). Comparisons with published sequences of known flaviviruses were performed by searches with FASTA program in EMBL database (available from www.ebi.ac.uk/embl/) to identify the detected agent and to study the level of homology.

One pool of *Cx. pipiens* captured in the middle of August 2006 from Delta del Llobregat, in a typical Mediterranean climate that contained 3 female mosquitoes, was positive for flaviviruses. That positive pool was obtained from the center of the village of Viladecans, where different common migratory and sedentary birds such as *Passer domesticus*, *Hirundo rustica,* or *Delichon urbica* feed and nest. The Spanish USUV sequence showed 97.97% homology to USUV strain SAAR-1776 from South Africa; it showed 94.94% similarity with USUV strain Vienna 2001 from Austria and USUV strain Budgapest from Hungary, with 2-nt and 5-nt differences, respectively (Figure). All of these were synonymous mutations and thus did not result in amino acids replacements. The homology data showed that the Spanish strain belongs to USUV species and is more related to the African USUV isolates than to central European isolates.

**Figure:**
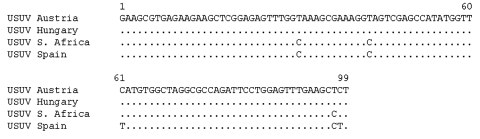
Comparison at nucleotide level of sequenced fragment among related Usutu virus (USUV). Dot indicates coincident nucleotide. The partial nucleotide sequence of detected Spanish USUV has been deposited in the GenBank database under accession no. AM909649. S. Africa, South Africa.

To date, no bird deaths observed in Barcelona Province have been associated with viral encephalitis. However, this region is where the USUV-specific RT-PCR–positive samples were obtained from *Cx. pipiens* mosquitoes. One possible explanation for these findings is that Spanish USUV could be naturally avirulent for birds because the African strains of USUV appear to be in Africa. Alternatively, USUV and other related viruses such as WNV may have been circulating in Spain for many years, as a result of regular reintroduction by birds migrating from Africa. Under such circumstances, natural genetic resistance, herd immunity, and cross-protective immunity caused by related viruses likely provided at least some protection against symptomatic infections. The discovery of USUV-specific RNA, most related to the African strains of USUV, in *Cx. pipiens* in Spain extends previous evidence (*7*,*10*) that USUV and related flaviviruses such as WNV are being introduced into western Europe from Africa, presumably by migratory birds.
